# Case Report: Atypical presentations of neonatal and infantile hemophilia B

**DOI:** 10.3389/fped.2026.1811557

**Published:** 2026-05-04

**Authors:** Arielle Locke, Nasrin Samji, Mihir Bhatt, Kay Decker, Sara J. Israels, Anthony K. C. Chan, Kyle Mendonça

**Affiliations:** 1 Division of Pediatric Hematology/Oncology, Department of Pediatrics, McMaster Children's Hospital, Hamilton, ON, Canada; 2 Department of Pediatrics, McMaster University, Hamilton, ON, Canada; 3 Department of Pediatric Hematology/Oncology, CancerCare Manitoba, Winnipeg, MB, Canada; 4 Department of Pediatrics and Child Health, University of Manitoba, Winnipeg, MB, Canada

**Keywords:** atypical presentation, congenital bleeding disorder, factor IX deficiency, hemophilia B, neonatal bleeding

## Abstract

Hemophilia B is an X-linked recessive bleeding disorder caused by deficiency or dysfunction of factor IX. It typically presents with spontaneous or trauma-induced bleeding, hemarthrosis, and soft tissue hematomas. We report three cases—two severe neonatal and one moderate infantile case—with atypical presentations: intra-abdominal hemorrhage with hepatic hematoma, a scrotal mass mimicking testicular torsion, and intracranial hemorrhage. None of the patients had a family history of bleeding disorders, and diagnoses were made after these significant bleeding episodes. These cases underscore the importance of considering congenital bleeding disorders in neonates and infants, especially males, who present with unexplained severe bleeding episodes in the absence of trauma or family history. Early recognition, preconception genetic counseling to identify the risk of bleeding disorders in parents, and individualized prophylactic approaches are essential to improve outcomes.

## Introduction

Hemophilia B is a rare inherited bleeding disorder caused by deficiency or dysfunction of coagulation factor IX, accounting for 15%–20% of all hemophilia diagnoses (1). It follows an X-linked recessive inheritance pattern, primarily affecting males; however, female carriers may also experience symptomatic bleeding and meet diagnostic criteria for hemophilia (1). While clinical manifestations in older children and adults are well described and typically include hemarthroses, hematomas, and trauma-induced bleeding, presentations in neonates and infants are less common and may be atypical (1–3).

Diagnosis in early life can be challenging, particularly when initial symptoms are non-specific, the family history is negative, or the bleeding presentation can be attributed to a more common etiology, such as birth trauma. We report three cases of moderate to severe hemophilia B with unusual and severe early-life presentations, emphasizing the need for increased clinical suspicion and prompt hemostasis laboratory evaluation in neonates and infants with unexplained bleeding.

## Case 1: Intraabdominal hemorrhage and hepatic hematoma

A 13 hour old boy, small for gestational age, presented with scrotal bruising, poor feeding, abdominal distension, lethargy, and hemodynamic instability. He was born via a non-traumatic spontaneous vaginal delivery to a primigravida mother with pregnancy-induced hypertension. There was no family history of bleeding disorders. His Apgar scores were 8 and 9 at 1 and 5 minutes, respectively. He was admitted to the neonatal intensive care unit (NICU) for evaluation of sepsis due to abdominal distension, lethargy, mottled skin, and hypoglycemia.

**Figure 1:**
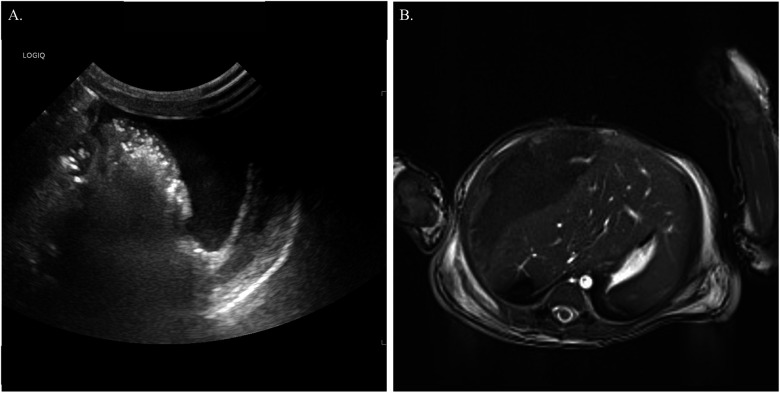
**(A)** Ultrasound abdomen: a single sonographic image of the left lower quadrant demonstrates a moderate amount of mildly complex fluid surrounding partially visualized bowel loops. **(B)** MRI liver: a single axial T2-weighted image of the upper abdomen demonstrates a moderate amount of heterogenous, predominantly hypointense fluid within the subcapsular anterolateral right hepatic lobe. The subcapsular hematoma exerts a moderate mass effect on the liver, with midline hepatic shift toward the left abdomen/left upper abdominal quadrant.

During transfer, he experienced a cardiac arrest requiring chest compressions, 10 minutes of positive pressure ventilation, and fluid resuscitation. Ultrasound revealed a right subcapsular hepatic hematoma with a possible liver laceration and a scrotal hematoma (Figure 1A). Coagulation studies indicated consumptive coagulopathy, and the overall clinical picture was consistent with disseminated intravascular coagulation (DIC). His laboratory investigations revealed a prolonged prothrombin time (PT) of 43.2 s (normal 10.1–12.8 s), elevated INR of 3.7 (normal 0.9–1.1), prolonged aPTT >150 s (normal 24.9–39.3 s), severely reduced fibrinogen of 0.4 g/L (normal 2–4.7 g/L), and markedly elevated D-dimer of 5307 ng/mL (normal <500 ng/mL). He required intensive supportive care, including transfusions of packed red blood cells (PRBCs), platelets, fresh frozen plasma (FFP), and fibrinogen during the first 10 days. Factor studies performed on day of life (DOL) 3–5 showed factor IX levels of 0.22, 0.19, and 0.27 U/mL, respectively; these were initially interpreted as secondary to consumptive coagulopathy in the setting of DIC. He developed oliguric acute kidney injury (Cr 227 μmol/L) and severe edema, necessitating continuous renal replacement therapy (CRRT) from DOL 4. Magnetic resonance imaging (MRI) on DOL 7 showed resolving hepatic hemorrhage without a clear etiology (Figure 1B). Cholestasis (direct bilirubin 294 μmol/L) was managed with triple phototherapy. CRRT was discontinued on DOL 11, and he was extubated by DOL 16. An ultrasound at discharge (DOL 31) showed a persistent subcapsular hepatic hematoma measuring 7.3 × 3.9 × 5.3 cm, although comparison with the earlier scan was limited due to uncertainty about the accuracy of the initial measurements. A follow-up ultrasound at 6 months of age showed a 1.7 cm amorphous calcification with a small subcapsular cyst, findings believed to represent sequelae of the prior hepatic laceration.

During the acute phase, no factor IX concentrate was administered, as the coagulopathy was attributed to DIC and managed with supportive care. Hemostatic investigations at 2 months of life identified severe hemophilia B (factor IX <0.01 U/mL). Genetic testing revealed pathogenic multiexon deletions involving F9 exons 7–8 and the 3′ end, consistent with the diagnosis. A plan for future treatment was initiated using on-demand recombinant factor IX (Eftrenonacog alfa, 500 units = 111 units/kg IV), with dosing aimed at achieving a target trough level of approximately 50%. He continues to receive treatment on an as-needed basis. Over 8 months of follow-up, he has experienced one minor, self-resolving episode of gum bleeding.

## Case 2: Scrotal mass mimicking torsion

A late preterm male developed a non-transilluminating scrotal swelling on DOL 9. He had a prior history of prolonged postvenipuncture bleeding following a septic workup for apnea on DOL 3; however, coagulation studies were not performed at the referring hospital. He received empiric antibiotics, which were later discontinued after negative blood cultures. He was born at 36 + 4 weeks' gestation to a healthy multiparous mother via elective cesarean section for maternal hypertension. Apgar scores were 9 and 9 at 1 and 5 minutes, respectively. Due to concern for potential testicular torsion, he was transferred to a tertiary care center.

**Figure 2:**
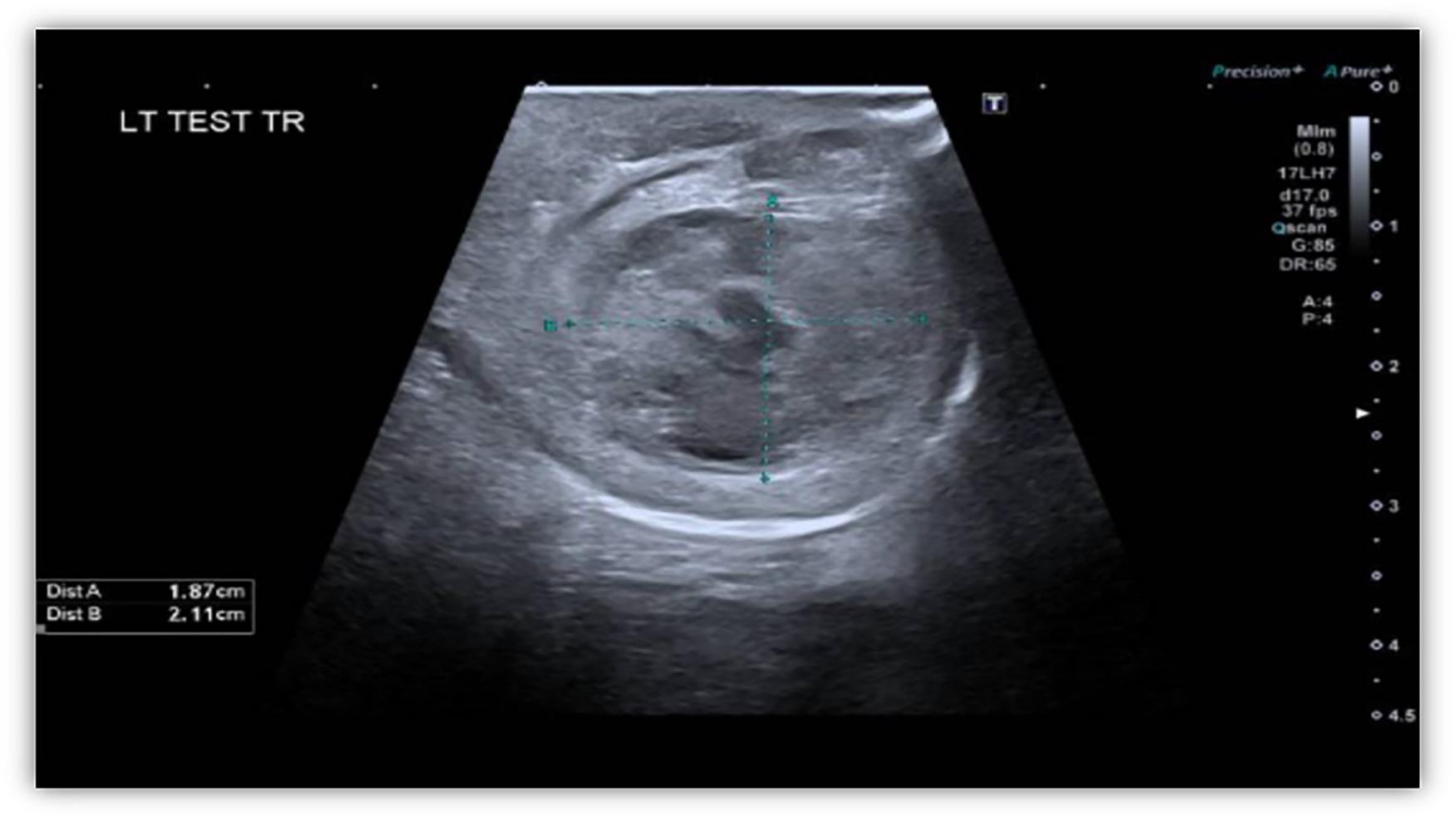
Doppler ultrasound of left testis: a single transverse grayscale sonographic image of the left testicle demonstrates a heterogeneous, predominantly echogenic lesion within the left testicular parenchyma. The echogenic lesion measures 1.9 cm × 2.1 cm × 2.5 cm. The differential diagnosis for the echogenic lesion includes hematocele, hematoma, abscess, or neoplasm.

The scrotal swelling was firm, purple, non-reducible, and tender. Doppler ultrasound on DOL 9 revealed a 2.1 × 1.9 × 2.5 cm echogenic lesion, raising concern for hematocele, hematoma, abscess, or neoplasm (Figure 2). On DOL 11, he developed an enlarging left antecubital hematoma at a venipuncture site, raising concern for brachial artery occlusion; however, perfusion remained intact. Additional bruising was noted on the penile shaft, left groin, scalp, and right foot. Coagulation studies on DOL 10 revealed a prolonged aPTT of 78 s (normal 25–41 s), with normal PT of 13.8 s (9.8–13.8 s), INR within the normal range (0.8–1.1), and fibrinogen of 2.4 g/L (normal 1.6–4.2). Factor IX level was <0.01 U/mL, confirming severe hemophilia B on DOL 11. Genetic testing was declined. No family history of bleeding disorders was reported, although the mother required an RBC transfusion following cesarean section.

He was treated with glycoPEGylated recombinant factor IX (Nonacog Beta Pegol, 250 units = 78 units/kg IV) on DOL 11 and 14, which increased the factor IX level to 0.56 U/mL 12 hours postinfusion. Prophylaxis with Nonacog Beta Pegol (1,000 units weekly) was initiated at 6 months following three traumatic bleeding episodes, and a port-a-cath was placed at 12 months. The factor IX dosing strategy aimed to maintain a trough factor IX activity of approximately 30%–50%, allowing safe outpatient management and reducing the need for frequent hospital visits. His lowest documented hemoglobin was 100 g/L at 9 months old, and he did not receive any PRBC transfusions during his clinical course. To facilitate therapy, his parents requested enrollment in a clinical trial evaluating a subcutaneous monoclonal antibody targeting the tissue factor pathway inhibitor.

## Case 3: Multicompartmental intracranial hemorrhage

A term male was born at 38 + 2 weeks’ gestation via elective repeat caesarean section. Delivery involved two vacuum pulls and pop-offs, but no bruising was noted. Apgar scores were 6 and 8 at 1 and 5 minutes, respectively. His multiparous mother had a history of intra-abdominal desmoid tumor treated with chemotherapy and a central-line-associated thrombosis managed with low-molecular-weight heparin, both during pregnancy. She also experienced diplopia early in pregnancy, attributed to estrogen, and a postpartum MRI revealed white matter lesions. She is currently being followed in a multiple sclerosis clinic. There is no family history of bleeding disorders, vascular abnormalities, or neurodegenerative disease. The infant's older sibling had duodenal atresia and VACTERL association.

On DOL 5, he was noted to have 16% weight loss, unconjugated hyperbilirubinemia, and lethargy. He was admitted to the NICU for intravenous hydration and phototherapy and discharged by DOL 8. No imaging was performed to rule out intracranial hemorrhage (ICH) as a cause of lethargy, and a coagulation screen was not obtained, despite a normal hemoglobin level at presentation. Parents reported to the NICU that he experienced prolonged postvenipuncture bleeding at his heel poke site, which was documented as not excessive and attributed to friction from kicking. This was not investigated further.

**Figure 3:**
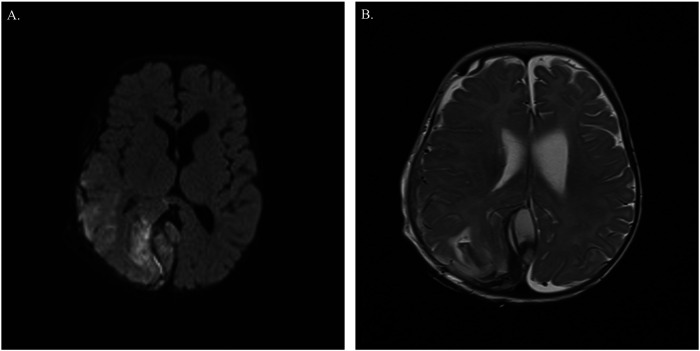
**(A)** CT brain: an axial unenhanced CT image of the brain shows a large multicompartmental intracranial hemorrhage (ICH), including subdural, subarachnoid, and possible epidural hemorrhage, along with right parietal–occipital infarcts, midline shift, and subfalcine herniation. **(B)** MRI brain: an axial T2-weighted MR brain image demonstrates heterogeneous extra-axial hemorrhage within the posterior interhemispheric fissure and over the right parieto-occipital and right frontotemporal lobes. A small amount of heterogeneous subarachnoid/intraparenchymal hemorrhage is also seen in the right parieto-occipital lobes. There is moderate edema in the right parieto-occipital lobes, with moderate mass effect on the right lateral ventricle, resulting in leftward midline shift.

At 4 months of age, he presented with an altered level of consciousness following a brief febrile illness. Physical examination revealed hypotonia, a bulging fontanelle, pinpoint pupils, and an increased head circumference. A CT scan of the head showed a large multicompartmental ICH, including subdural, subarachnoid, and possible epidural hemorrhage, along with right parietal–occipital infarcts, midline shift, and subfalcine herniation (Figure 3A). He underwent an emergent right hemicraniectomy and intracranial pressure monitor placement. Intraoperatively, he received PRBC transfusion (10 mL/kg) and platelet transfusion (10 mL/kg). Coagulation studies obtained following emergency administration of FFP (10 mg/kg) revealed a prolonged aPTT of 44.4 s (normal 24.9–39.3 s), PT of 12.8 s (normal 10.1–12.8 s), INR of 1.1 (normal 0.9–1.1), and fibrinogen of 1.8 g/L (normal 2–4.7 g/L). Factor IX level measured after FFP administration was 0.04 IU/mL, confirming hemophilia B. He received 1,000 units (130 units/kg) of recombinant factor IX (Eftrenonacog alfa) every 2 days for 8 days, achieving trough factor IX levels of approximately 50%. A port-a-cath was placed, and he was started on recombinant factor IX/Eftrenonacog alfa 500 units (66 units/kg) twice weekly.

Five weeks later, he underwent autologous cranioplasty, during which an accidental subclavian vein catheterization occurred without bleeding complications, as it was managed with factor IX replacement. Ophthalmologic evaluation revealed a left homonymous hemianopia. He remains on levetiracetam for seizure prophylaxis and continues to be monitored by physical and occupational therapy, with no developmental concerns or further bleeding episodes to date. Genetic testing identified a pathogenic *F9* variant (Gly106Asp); maternal genotyping did not detect this variant, indicating a *de novo* mutation in this patient.

## Discussion

These three cases illustrate the variable and unpredictable clinical spectrum of bleeding in neonates and infants with hemophilia B, ranging from isolated hematomas to life-threatening systemic hemorrhage. While severe hemophilia B is typically associated with spontaneous bleeding, these cases demonstrate that factor IX deficiency can present in very young patients with diverse and atypical manifestations before a diagnosis is established. It is also important to note that neonates, especially preterm infants, have lower physiological factor IX levels, which can complicate early definitive diagnoses of hemophilia B, highlighting the importance of early involvement of pediatric hematology in such cases (4).

The first case involved intra-abdominal hemorrhage and hepatic hematoma within the first hours of life, in the absence of delivery trauma, highlighting the potential for spontaneous bleeding and diagnostic confusion with sepsis or DIC. Notably, a recent similar case report described spontaneous hepatic rupture in a term 2-day-old neonate (5). The second case presented as a scrotal mass initially concerning for testicular torsion. However, the presence of extensive bruising and postvenipuncture bleeding led to the diagnosis of severe hemophilia B. This presentation parallels a report by Lee et al., in which a scrotal hematoma was the first clinical manifestation of severe hemophilia A, suggesting the importance of considering bleeding disorders in the differential diagnosis of acute scrotal swelling (6). The third case describes a child who developed spontaneous multicompartmental ICH at 4 months of life, in the absence of prior bleeding or family history of a bleeding disorder. This case highlights the challenges of diagnosing a bleeding disorder without a known family history, the potential implications of delayed recognition, and the need to consider hemophilia in infants presenting with unexplained neurologic deterioration. The identification of a *de novo* pathogenic *F9* variant underscores the genetic complexity and reinforces the need for genetic counseling, even in sporadic cases. This case is consistent with a CDC report by Kulkarni et al., who reported that approximately 7% of infants with hemophilia experience ICH within the first 2 years of life, often due to delivery trauma or spontaneous bleeding in the absence of trauma (7).

Other atypical presentations reported in the literature include hemorrhagic shock after fetal scalp blood sampling, intrahepatic bleeding with intracerebral thrombosis, mandibular pseudotumor in infancy, upper airway hemorrhage, trauma-induced splenic lesions, gastrointestinal hemorrhage, and spontaneous bleeding causing acute torticollis in older children, further highlighting the clinical spectrum of hemophilia B (8–15). Although none of our cases had a family history of bleeding disorders, their initial presentations with unexplained bleeding/bruising highlight the importance of considering congenital bleeding disorders in neonates and infants, especially males with unexplained coagulopathy, irrespective of delivery or family history.

Early initiation of factor IX replacement was key in achieving hemostatic stability in all three cases, preventing the progression of bleeding and reducing the risk of further life-threatening complications. Only two of the three patients received early prophylaxis, while Case 1 remained on on-demand therapy at the family's request. Results from the Paradigm 5 trial support the use of once-weekly Nonacog Beta Pegol in young children, demonstrating mean trough levels of 15%–19% with excellent hemostatic control (16). Although targets in Case 2 were higher, this approach aligns with the principles of individualized prophylaxis, which allow for higher goals in neonates and infants with faster clearance, complex presentations, or the need to minimize venous access. Case 2 also received a higher-than-standard prophylactic dose, which was used as part of an individualized approach based on his bleeding phenotype and clinical needs. Timely administration of factor IX not only stabilized acute hemorrhagic events but also facilitated coordinated multidisciplinary care, including critical care, hematology, surgery, and rehabilitation. These outcomes reinforce the importance of early recognition and intervention in suspected hemophilia, as delays in factor replacement can significantly impact morbidity. The use of extended half-life factor IX products, combined with the exploration of clinical trials, demonstrates advancements in hemophilia management strategies aimed at reducing treatment burden and improving patient outcomes.

Early recognition, genetic counseling, and personalized prophylaxis are crucial to optimize outcomes and prevent life-threatening complications. Genetic counseling improves outcomes by enabling accurate molecular diagnosis, early identification of at-risk relatives, and informed reproductive planning (17–19). It also provides essential psychosocial support, helping families understand inheritance patterns and make informed decisions across generations (20). Optimally, hemostasis laboratory investigations should be performed prior to the administration of blood products to ensure an accurate diagnosis and appropriate management. These cases emphasize the need for pediatric awareness of the diverse presentations of hemophilia B across pediatric age groups and support the broader clinical imperative to include congenital bleeding disorders in the differential diagnosis of unexplained hemorrhage in infancy.

## Data Availability

The original contributions presented in the study are included in the article/Supplementary Material, further inquiries can be directed to the corresponding author.

## References

[1] Srivastava A Santagostino E Dougall A Kitchen S Sutherland M Pipe SW WFH Guidelines for the management of hemophilia, 3rd edition. Haemophilia. (2020) 26(S6):1–158. 10.1111/hae.14046 32744769

[2] Chalmers EA. Haemophilia and the newborn. Blood Rev. (2004) 18(2):85–92. 10.1016/S0268-960X(03)00062-6 15010147

[3] Chalmers E Williams M Brennand J Liesner R Collins P Richards M. Guideline on the management of haemophilia in the fetus and neonate. Br J Haematol. (2011) 154(2):208–15. 10.1111/j.1365-2141.2010.08545.x 21554256

[4] Neary E Mccallion N Kevane B Cotter M Egan K Regan I Coagulation indices in very preterm infants from cord blood and postnatal samples. J Thromb Haemost. (2015) 13(11):2021–30. 10.1111/JTH.13130 26334448

[5] Liu S Wang R-Y Wang C-T. Hepatic rupture in a neonate with hemophilia B: a case report. J Pediatr Surg Case Reports. (2025) 123:103137. 10.1016/J.EPSC.2025.103137

[6] Lee JS-Y Chieng C-H Martin M Toh T-H. Spontaneous neonatal scrotal haematoma: an early manifestation of severe haemophilia. BMJ Case Rep. (2021) 14(4):e241482. 10.1136/bcr-2020-241482 33910804 PMC8094324

[7] Kulkarni R Presley RJ Lusher JM Shapiro AD Gill JC Manco-Johnson M Complications of haemophilia in babies (first two years of life): a report from the Centers for Disease Control and Prevention Universal Data Collection System. Haemophilia. (2017) 23(2):207–14. 10.1111/hae.13081 27813214 PMC5354941

[8] Sabir H Stannigel H Schwarz A Hoehn T. Perinatal hemorrhagic shock after fetal scalp blood sampling. Obstet Gynecol. (2010) 115(2 Part 2):419–20. 10.1097/AOG.0b013e3181c51aeb 20093865

[9] Douvas MG Monahan PE. Life-threatening thrombosis complicating the management of hepatic hemorrhage: anticoagulant treatment in a newborn with hemophilia B. J Pediatr Hematol Oncol. (2004) 26(4):258–63. 10.1097/00043426-200404000-00010 15087956

[10] Cox DP Solar A Huang J Chigurupati R. Pseudotumor of the mandible as first presentation of hemophilia in a 2-year-old male: a case report and review of jaw pseudotumors of hemophilia. Head Neck Pathol. (2011) 5(3):226–32. 10.1007/s12105-011-0267-x 21567186 PMC3173544

[11] Bray G Nugent D. Hemorrhage involving the upper airway in hemophilia. Clin Pediatr (Phila). (1986) 25(9):436–9. 10.1177/000992288602500903 3742923

[12] Koren JP Klein RL Kavic MS Krill CEJ. Management of splenic trauma in the pediatric hemophiliac patient: case series and review of the literature. J Pediatr Surg. (2002) 37(4):568–71. 10.1053/jpsu.2002.31611 11912512

[13] Park SH Choe BH. Moderate hemophilia B diagnosed by massive gastrointestinal hemorrhage on the first day of life: a case report and literature review. Neonatal Med. (2016) 23(4):238–41. 10.5385/nm.2016.23.4.238

[14] Sankar J Srinivasan A Ramakrishnan V Balasubramaniam C. An unusual cause of acute torticollis. Clin Pediatr (Phila). (2009) 48(7):784–5. 10.1177/0009922808330785 19224866

[15] Reish O Nachum E Naor N Ghoshen J Merlob P. Hemophilia B in a neonate: unusual early spontaneous gastrointestinal bleeding. Am J Perinatol. (1994) 11(3):192–3. 10.1055/s-2008-1040743 8048983

[16] Carcao M Zak M Abdul Karim F Hanabusa H Kearney S Lu M-Y Nonacog beta pegol in previously treated children with hemophilia B: results from an international open-label phase 3 trial. J Thromb Haemost. (2016) 14(8):1521–9. 10.1111/jth.13360 27174727

[17] Sivapalaratnam S Collins J Gomez K. Diagnosis of inherited bleeding disorders in the genomic era. Br J Haematol. (2017) 179(3):363–76. 10.1111/BJH.14796 28612396

[18] Vidal F. State of the art of genetic studies in hemophilia carriers. Blood Coagul Fibrinolysis. (2020) 31(1S):S4–5. 10.1097/MBC.0000000000000992 33351493

[19] García-Lozano JC Lozano-Arana MD. Prenatal diagnostic techniques and IVF in patients with coagulopathies. Blood Coagul Fibrinolysis. (2020) 31(1S):S6–8. 10.1097/MBC.0000000000000991 33351494

[20] Miller R. Counselling about diagnosis and inheritance of genetic bleeding disorders: haemophilia A and B. Haemophilia. (1999) 5(2):77–83. 10.1046/J.1365-2516.1999.00288.X 10215953

